# Negative ^18^F-FET PET/CT in brain metastasis recurrence: a teaching case report

**DOI:** 10.1186/s41824-021-00115-0

**Published:** 2021-11-22

**Authors:** Samirah Alshehri, John Prior, Mohammed Moshebah, Luis Schiappacasse, Vincent Dunet

**Affiliations:** 1 Service of Radiation Oncology, Lausanne University Hospital and University of Lausanne, Lausanne, Switzerland; 2 Service of Nuclear Medicine and Molecular Imaging, Lausanne University Hospital and University of Lausanne, Lausanne, Switzerland; 3 Service of Diagnostic and Interventional Radiology, Lausanne University Hospital and University of Lausanne, Lausanne, Switzerland

**Keywords:** Radiation, Necrosis, Brain, Metastasis

## Abstract

Positron emission tomography (PET) using O-(2-[^18^F]fluoroethyl)-L-tyrosine (^18^F-FET) PET has been shown to be a useful tool for differentiating radiation therapy outcomes, such as brain metastasis recurrence or radiation necrosis. We present the case of a female patient with brain metastases from pulmonary mucinous adenocarcinoma with suspicion of tumor recurrence on follow-up magnetic resonance imaging (MRI) after radiosurgery. ^18^F-FET PET/computed tomography (CT) was indicative of radiation necrosis. Due to the patient's medical history and the discrepancy between the brain MRI and PET/CT results, surgical biopsies were decided, which were positive for brain metastasis recurrence. The diagnosis of metastasis recurrence may also be challenging on ^18^F-FET PET/CT. In case of discrepancies between MRI and PET/CT results, false-negative ^18^F-FET PET/CT remains a possibility and requires careful follow-up or biopsy.

## Introduction

O-(2-[^18^F]fluoroethyl)-L-tyrosine PET (^18^F-FET) is an artificial amino acid taken up by upregulated tumor cells but not incorporated into proteins. ^18^F-FET positron emission tomography/computed tomography (PET/CT) is increasingly used in daily practice, especially in the imaging of primary brain tumors and metastatic lesions. It demonstrated very good performance for the initial assessment of patients with new, isolated, untreated brain lesions (Dunet et al. 2012). It has also proven to be useful for the characterization of post-radiotherapy lesion changes in a few retrospective studies, mainly limited by the mixed histological population and absence of pre-therapy imaging. We present a case of false-negative ^18^F-FET PET/CT examination in a patient with treated brain metastases from pulmonary mucinous adenocarcinoma. Treated metastases had low to moderate tracer avidity, and quantitative metrics were consistent with radionecrosis. There were initial concerns regarding the discordance between PET/CT and magnetic resonance imaging (MRI), which showed signs of tumor recurrence. Surgical biopsies were decided after multidisciplinary discussions and showed the lesion corresponding to the tumoral tissue residue. This case illustrates that published cut-off values of ^18^F-FET parameters might not be appropriate for evaluating treated brain metastases from every cancer type in daily practice.

## Case report

A 58-year-old woman with cerebral metastases from lung cancer of the right upper lobe presented with the progression of lesion size on follow-up MRI. She was initially diagnosed with mucinous adenocarcinoma 4 years previously, which was confirmed after a bronchoscopic biopsy and staged cT1a (0.6 cm) cN2 (station 4R) cM1c (five brain metastases), stage IVB (according to the TNM 8th edition). Histopathological analyses demonstrated no EGFR, ERBB2, or BRAF mutations, no ALK/ROS1 rearrangement, or PD-L1 expression, but KRAS (G12S, exon 2) and TP53 (C275S, exon 8) mutations. Initial cerebral MRI findings were suggestive of metastatic disease.

The primary lung tumor was treated with radio-chemotherapy, which included four cycles of cisplatin-pemetrexed from January 2018 to March 2018 and concomitant radiotherapy in the right upper lobe and mediastinum with a total dose of 66 Gy (i.e., 33 fractions of 2 Gy) from February 2018 to March 2018. The brain metastases were treated in October 2018 with stereotactic radiotherapy (SRT) at a dose of 20 Gy in a single fraction. Two new metastases in the left frontal lobe (December 2018) and right frontal lobe (March 2019) were subsequently treated with SRT with a single dose of 20 Gy.

**Fig. 1:**
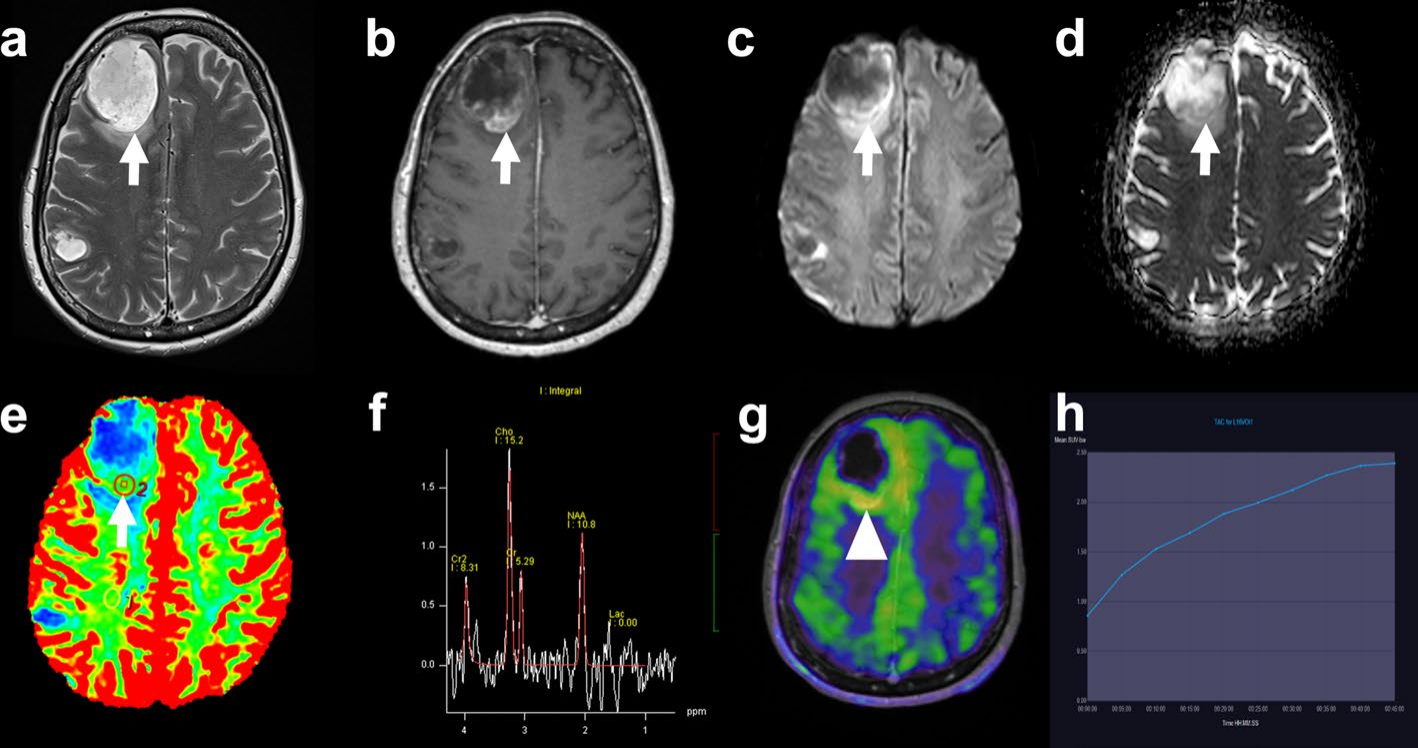
MRI and 18F-FET PET/CT findings. In our 58-year-old female patient, a follow-up brain MRI performed 16 months after radiosurgery showed an increase in the size of a treated right frontal lesion (white arrow) that appeared hyperintense on T2 weighted images (**a**), with central necrosis and peripheral thick contrast enhancement on T1 weighted images (**b**). The periphery of the lesion was bright on diffusion-weighted imaging (**c**) with moderately low ADC (**d**). On perfusion-weighted images, the nrCBV was 2.3 (**e**) and nrCBF was 2.8 (not shown) while MR spectroscopy (**f**) showed a high peak of choline with Cho/Cr ratio of 2.9, which overall indicated tumor residue. While 18F-FET PET/CT raw images showed moderate peripheral uptake (**g**, arrowhead), the maximum target-to-background ratio was 2.2 (i.e. lower than 2.55) and the time-activity-curve showed increasing uptake over time (**h**), which was interpreted as signs of radiation necrosis

The patient was followed up with MRI scans every 2–3 months. In November 2019, the follow-up MRI showed an increase in the size of two treated lesions, one in the right frontal lobe and the other in the right parietal lobe, suggestive of radionecrosis, and a 2-month control was recommended according to the multidisciplinary tumor board. The subsequent MRI showed an increase in the size of the right frontal lesion with an enhancing soft tissue component in its posterior part, whose perfusion (nrCBV = 2.3, nrCBF = 2.8) and spectroscopy (Cho/Cr ratio = 2.9) parameters suggested the persistence of tumor residue in this region (Fig. 1).

^18^F-FET PET/CT was performed to further characterize these changes and showed a heterogeneous and low to moderate uptake of the radiotracer at the level of the right frontal lesion. The maximum tumor-to-brain ratio (TBRmax = lesion SUVmax divided by normal contralateral brain SUVmean) was 2.2 while the time-activity curve (TAC) displayed a cumulative pattern, a time-to-peak of > 40 min and a late phase slope of 0.178 SUV/h (estimated by linear regression on the 20–50 min post-injection frames) (Ceccon et al. 2017), which was overall in favor of a radionecrotic lesion (Fig. 1). No significant radiotracer uptake was observed in the other metastatic lesions treated with radiosurgery.

In this context, her case was re-discussed at the multidisciplinary brain metastases board, where surgical resection of the right frontal lesion was proposed.

Histopathological analyses of excisional biopsies of the lesion confirmed metastatic recurrence of her initial mucinous adenocarcinoma, while the dura mater biopsies showed no metastatic cells; however, revealed chronic calcified inflammatory reaction with foreign bodies.

## Discussion

A current issue frequently encountered in the oncological management of brain metastases is differentiating tumor recurrence from treatment-related changes following stereotactic radiation therapy. Sometimes brain MRI does not always allow a clear differentiation between local brain tumor recurrence and progression from radiation-induced changes, including radiation necrosis. Radiation necrosis usually manifests within 6–12 months after radiotherapy treatment and occurs in approximately 5%–25% of all treated patients (Kumar et al. 2000; Shah et al. 2013). A similar rate is found in patients with brain metastases treated with radiosurgery (Minniti et al. 2011), taking into consideration that the rate could change according to the radiation total dose, field, number, and frequency of doses.

On MRI, a common characteristic of radiation necrosis on contrast-enhanced sequences is the geographic enhancement or Swiss cheese-like enhancement (Kumar et al. 2000). Nonetheless, conventional MRI is not always sufficient to differentiate tumor progression/recurrence from treatment-related effects (Mullins et al. 2005). Some sequences may have additive value in oncological imaging. Diffusion-weighted imaging, especially the apparent diffusion coefficient (ADC) sequences, can help differentiate tumor recurrence and radiation necrosis. Because of the low water mobility properties in high cellular lesions, the ADC will be low in the case of tumor recurrence. In contrast, an increased ADC is attributed to increased water mobility in cases of radiation necrosis (Asao et al. 2005). In addition, magnetic resonance spectroscopy (MRS) is an ongoing subject of research and may show that N-acetyl aspartate (NAA) and creatinine (Cr) decrease in cases of radiation necrosis, whereas high choline (Cho) levels are correlated with tumor recurrence (Rock et al. 2004; Sundgren et al. 2006). The Cho/Cr and Cho/NAA ratios have been described as good markers for differential diagnosis (Dowling et al. 2001; Plotkin et al. 2004). MR perfusion techniques using contrast enhancement can measure the relative cerebral blood volume (rCBV) and estimate vascularity and hemodynamics. Hyperperfusion is seen in tumor progression, and hypoperfusion is observed in radiation necrosis (Aronen and Perkio 2002; Ellika et al. 2007). It has been reported that rCBV values < 0.6 suggest radiation necrosis and values > 2.6 suggest tumor progression (Sugahara et al. 2000).

Several PET/CT tracers have been investigated as imaging modalities to distinguish the treatment effect from tumors in clinical practice (Galldiks et al. 2019). Among them, ^18^F-FET PET showed a high diagnostic performance using tumor-to-brain ratios and dynamic parameters with a sensitivity of 95% and specificity of 91% (Galldiks et al. 2012). Presently, the differentiation of radiation injury from metastasis recurrence using amino acid PET has been the most thoroughly investigated indication (Galldiks et al. 2019), repeatedly demonstrating high diagnostic accuracy. Galldiks et al. reported that the combined evaluation of the TBRmean of ^18^F-FET uptake and the pattern of the TAC can differentiate local brain metastasis recurrence from radionecrosis with high accuracy (Galldiks et al. 2012). This study and others were confirmed by Ceccon et al., who reported that the optimal cut-off value was a TBRmax > 2.55 (sensitivity = 83%, specificity = 85%), TBRmean > 1.95 (sensitivity = 86%, specificity = 88%), time-to-peak < 32.5 min (sensitivity = 58%, specificity = 73%), and TAC slope < 0.125 SUV/h (sensitivity = 68%, specificity = 61%) for identification of recurrence (Ceccon et al. 2017). It is worth mentioning that these results were obtained mainly from retrospective analyses in small cohorts and that there was no histological confirmation of the diagnosis in many cases. In addition, it remains poorly known whether all metastases from different primary tumors behave the same regarding ^18^F-FET uptake at baseline or during follow-up, which could limit the reproducibility of these preliminary reports. Some authors have reported a wide range of lesion uptake in either primary solid tumors or related brain metastases (Galldiks et al. 2012; Unterrainer et al. 2017; Pauleit et al. 2005). In particular, as in our case, adenocarcinoma from various organs often demonstrates low ^18^F-FET uptake (Galldiks et al. 2012; Pauleit et al. 2005). Other potential confounding factors should also be considered when using quantitative metrics such as tumor genotype, intratumoral hemorrhage, corticoid intake (Stegmayr et al. 2019), or immunotherapy and targeted therapy (Galldiks et al. 2021), which could modify the lesion, microenvironment, and normal brain uptake. Finally, one point to be considered in the differential diagnosis is that very small lesions in which the SUV may not be sufficient to reach the threshold value of 2.55 due to the partial volume effect. Further histological correlation and large prospective studies are now needed, especially to optimize ^18^F-FET uptake and TAC slope cut-off values according to primary tumor types to detect progression and optimize patient management.

## Conclusions

In conclusion, the causes of false-negative ^18^F-FET uptake have not been well investigated, and actual evidence of this in the literature is scarce. This case demonstrates important teaching points for the tumor board team involved in the management of patients with brain metastases. In the event of inconsistent findings between MRI and ^18^F-FET PET/CT, care must be taken before the final diagnosis, especially for pulmonary mucinous adenocarcinoma. Collaborative discussion between radio-oncologists, radiologists, and nuclear physicians can help favor one diagnosis over the other. Another point is that although ^18^F-FET PET/CT is sensitive and specific for detecting post-treatment changes in brain metastases, it is important to be careful about the pattern of lesion uptake, as in our case.

## Data Availability

All data generated or analysed during this study are included in this published article.

## Abbreviations

ADC: Apparent diffusion coefficient
Cho: Choline
Cr: Creatinine
^18^F-FET: O-(2-[^18^F]fluoroethyl)-L-tyrosine
MRS: Magnetic resonance spectroscopy
NAA: *N*-Acetyl aspartate
PET/CT: Positron emission tomography/ computed tomography
rCBV: Relative cerebral blood volume
TAC: Time activity curve
TBR: Tumor-to-brain ratio
